# Multi-Omics Strategies to Investigate the Biodegradation of Hexahydro-1,3,5-trinitro-1,3,5-triazine in *Rhodococcus* sp. Strain DN22

**DOI:** 10.3390/microorganisms12010076

**Published:** 2023-12-30

**Authors:** Xiangzhe Zhou, Qifa Yao, Nuomin Li, Min Xia, Yulin Deng

**Affiliations:** 1 School of Medical Technology, Beijing Institute of Technology, Beijing 100081, China; 3120211440@bit.edu.cn (X.Z.);; 2 School of Materials Science and Engineering, Beijing Institute of Technology, Beijing 100081, China

**Keywords:** RDX degradation, *Rhodococcus* sp. strain DN22, genome sequencing, proteomics, metabolomics

## Abstract

Hexahydro-1,3,5-trinitro-1,3,5-triazine (RDX) is an energetic and persistent explosive with long-lasting properties. *Rhodococcus* sp. strain DN22 has been discovered to be a microbe capable of degrading RDX. Herein, the complete genome of *Rhodococcus* sp. strain DN22 was sequenced and analyzed. The entire sequences of genes that encoded the two proteins participating in RDX degradation in *Rhodococcus* sp. strain DN22 were obtained, and were validated through proteomic data. In addition, few studies have investigated the physiological changes and metabolic pathways occurring within *Rhodococcus* sp. cells when treated with RDX, particularly through mass spectrometry-based omics. Hence, proteomic and metabolomic analyses were carried out on *Rhodococcus* sp. strain DN22 with the existence or lack of RDX in the medium. A total of 3186 proteins were identified between the two groups, with 115 proteins being significantly differentially expressed proteins. There were 1056 metabolites identified in total, among which 130 metabolites were significantly different. Through the combined analysis of differential proteomics and metabolomics, KEGG pathways including two-component system, ABC transporters, alanine, aspartate and glutamate metabolism, arginine biosynthesis, purine metabolism, nitrogen metabolism, and phosphotransferase system (PTS), were observed to be significantly enriched. These findings provided ponderable perspectives on the physiological alterations and metabolic pathways in *Rhodococcus* sp. strain DN22, responding to the existence or lack of RDX. This study is anticipated to expand the knowledge of *Rhodococcus* sp. strain DN22, as well as advancing understanding of microbial degradation.

## 1. Introduction

Hexahydro-1,3,5-trinitro-1,3,5-triazine (RDX), a kind of nitramine explosive, has detrimental effects on both aquatic and terrestrial life forms [1]. Despite being stable and persistent in the environment [2], RDX has been observed to undergo degradation by various microorganisms [3]. Since the last century, numerous strains have been discovered to be capable of degrading RDX, including *Rhodococcus* sp. strain DN22 [4], *Rhodococcus rhodochrous* strain 11Y [5], *Williamsia* sp. KTR4 and *Gordonia* sp. KTR9 [6], *Pseudomonas fluorescens* I-C, and *Pseudomonas putida* II-B [7], among others. *Rhodococcus* members display an extraordinary capability to degrade xenobiotic compounds through diverse catabolic pathways [8]. Certain substances, such as 4-nitro-2,4-diazbutanal (NDAB), methylenedinitramine (MEDINA), and HCHO, have been determined as proposed products in RDX degradation [9,10,11]. *Rhodococcus* sp. strain DN22 and *Rhodococcus rhodochrous* strain 11Y could degrade RDX through denitration, followed by ring cleavage, producing nitrite, formaldehyde, and 4-nitro-2,4-diazabutanal [9,12] under aerobic conditions [13]. Furthermore, it is worth mentioning that a three-year field trial demonstrated the in situ efficacy of RDX biodegradation by a switchgrass transgenic from *Rhodococcus rhodochrous* strain 11Y [14].

Via bioinformatics analysis, it was found that the microbe community structure and the metabolomics features in soil treated with RDX were altered [15]. In addition, shotgun sequencing was applied to investigate the diversity and abundance of organisms and genes related to RDX degradation in groundwater before and after biostimulation [16]. A recent study provided a detailed description of Actinomycetes species isolated from water, wastewater, and sludge, degrading RDX and 2,4,6-trinitrotoluene [17]. Utilizing microarray hybridizations, transcriptomics was performed to investigate the character of nitrogen limitation in RDX biodegradation by *Gordonia* sp. strain KTR9 [18]. Nevertheless, little research has investigated the physiological changes and metabolic pathways occurring within *Rhodococcus* sp. cells when treated with RDX, particularly through mass spectrometry-based omics. In this study, multi-omics were utilized in *Rhodococcus* sp. strain DN22 to excavate more valuable biochemical information. Previous research has suggested that a cytochrome P450 enzyme participates in RDX degradation in *Rhodococcus* sp. strain DN22 [19]. Homologues (>99%) of determined genes, which encoded proteins (cytochrome P450-like protein XplA and adrenodoxin reductase-like) involved in RDX degradation in *Rhodococcus rhodochrous* strain 11Y [5], were amplified from *Rhodococcus* sp. strain DN22 [20]. Despite respectable studies conducted on *Rhodococcus* sp. strain DN22, the proteins participating in RDX degradation in the strain remain fragments. Here, the complete genome of *Rhodococcus* sp. strain DN22 was sequenced and analyzed, and the entire sequences of genes that encoded the two proteins involved in RDX degradation in *Rhodococcus* sp. strain DN22 were acquired, and were validated through proteomic data. Additionally, differential proteomics and metabolomics were performed on *Rhodococcus* sp. strain DN22 with or without RDX in the medium, extending the understanding of RDX biodegradation from a new perspective. The combined analysis revealed that several KEGG pathways with important biological significance were significantly enriched. This study has provided a more holistic insight into *Rhodococcus* sp. strain DN22. These investigations are anticipated to expand the knowledge of microbial degradation.

## 2. Materials and Methods

### 2.1. Bacterial Growth Conditions and RDX Analytical Method

*Rhodococcus* sp. strain DN22 was originally isolated from polluted soil [4] and kindly provided by Dr. Nicholas Coleman. Bacteria were incubated aerobically in liquid culture at 25 °C [4], shaking in 100 mL of sterilized medium in 300 mL Erlenmeyer flasks at 160 r/min. LB medium was used as a normal medium. A medium utilizing RDX as an exclusive nitrogen source was prepared using Wahaha purified water with 40 mg/L RDX and a minimal medium. The minimal medium consisted of 5 mmol/L glucose, 5 mmol/L succinate, 10 mmol/L glycerol, 11.39 mmol/L KH_2_PO_4_, 28.42 mmol/L K_2_HPO_4_ [21], and trace element solution (1% *v*/*v*). The trace element solution comprised 50 mL/L HCl, 0.062 g/L H_3_BO_3_, 0.25 g/L CuSO_4_·5H_2_O, 0.28 g/L CoSO_4_·7H_2_O, 1.11 g/L MnSO_4_·4H_2_O, 1.44 g/L ZnSO_4_·7H_2_O, 2 g/L CaCO_3_, 5.6 g/L FeSO_4_·7H_2_O, and 61 g/L MgSO_4_·7H_2_O (slightly modified from the previous method [22]). The growth of *Rhodococcus* sp. strain DN22 was measured by the absorbance at optical densities of both 530 nm (OD_530_) [4] and 600 nm (OD_600_) [21].

RDX was examined through high-performance liquid chromatography (HPLC) from Shimadzu. Samples (1 mL) were centrifuged at 10,000× *g* for 1–5 min at 25 °C. Supernatants were filtered (0.22 μm) into sample vials. An amount of 5 μL of the samples was separated on a C_18_ chromatographic column (Dikma Spursil, 5 μm, 150 × 3.0 mm) at a column oven temperature of 25 °C. An isocratic mobile phase comprising 40% CH_3_OH and 60% H_2_O was utilized, and delivered at a flow rate of 0.400 mL/min, with detection at 232 nm [4].

### 2.2. Bacterial Growth and RDX Degradation Detection

The growth curve of *Rhodococcus* sp. strain DN22 cultured in LB medium was initially determined, to identify its logarithmic phase for complete genome sequencing, as well as its early stationary phase, in which the bacteria were harvested and inoculated into the medium utilizing RDX as an exclusive nitrogen source [4]. The experiment was performed in triplicate. The bacterial solution was taken periodically for OD_530_ as well as OD_600_ detection.

To ascertain the bacterial growth curve and RDX degradation by the bacteria in the medium utilizing RDX as an exclusive nitrogen source, bacteria were initially cultured in LB medium until the early stationary phase. Subsequently, bacteria were collected through centrifugation (4000 r/min, 10 min) and washed thrice in PBS (4000 r/min, 10 min). Approximately 20 mg of bacterial cells in PBS were inoculated into the medium utilizing RDX as an exclusive nitrogen source. The experiment was conducted in triplicate with the RDX-free medium as the control. Bacterial solution samples were taken at intervals, for OD_530_ and OD_600_ detection, as well as RDX level analysis.

### 2.3. Complete Genome Sequencing

#### 2.3.1. Bacterial Cell Pellet Preparation

*Rhodococcus* sp. strain DN22 was cultured in LB medium for one day. Bacteria were collected through centrifugation (3500 r/min, 10 min) and washed with PBS (3500 r/min, 10 min). The cells were frozen swiftly with liquid N_2_ and subsequently kept at a temperature of −80 °C, pending DNA extraction.

#### 2.3.2. DNA Extraction and Library Preparation

Short-read second-generation DNA extraction and long-read third-generation DNA extraction (column-based) were performed, providing high-quality DNA for library preparation. For the short-read second-generation, whole genome sequencing library preparation was carried out, involving sample quality control, DNA fragmentation, fragment selection, end repair, 3′ adenylating, adaptor ligation, PCR, library quality control, and circularization. For the long-read third-generation, bacterial barcode library preparation was completed by sample quality control, g-TUBE fragmentation, enzymatic digestion, damage repair, end repair, barcode ligation, pooling, BluePippin sorting, and library quality control.

#### 2.3.3. Genome Sequencing and Assembly

The DNBSEQ and PacBio Sequel II platforms at the Beijing Genomics Institute were utilized for genome sequencing of *Rhodococcus* sp. strain DN22. The PacBio platform employed four SMRT cells *Zero-Mode Waveguide* arrays of sequencing, for subread set generation.

Data filtering was performed before genome assembly. For the DNBSEQ data, the filtering process involved removing reads with a certain proportion of low-quality (≤20) bases (40% as default) and of Ns (0.1% as default), as well as removing adapter and duplication contamination. For the PacBio Sequel II data, subreads were extracted from polymerase reads, and subreads less than 1000 bp were excluded. Highly accurate circular consensus sequencing (CCS) data were obtained from subreads by SMRT Analysis. The parameters for SMRT Analysis (v2.3.0) were set as follows: estn = 24, nproc = 8, cov = 6.

Various software was utilized for genome assembly, and the process included subread correction, corrected read assembly, correction of assembly result, judgment for sequence loop, and discrimination between chromosome and plasmid sequences [23,24,25,26,27]. Canu and Falcon were used to obtain highly reliable corrected reads in subread correction. Canu and Falcon were employed, respectively, to ultimately acquire the best assembly result in corrected read assembly. The parameters for Canu (v1.5) were set as follows: estn = 24, npruseGrid = 0, corOvlMemory = 4. The parameters for Falcon (v0.3.0) were set as follows: -v -dal8 -t32 -h60 -e.96 -l500 -s100 -H3000. GATK was utilized to perform single-base corrections with second-generation sequencing data in the correction of assembly result, enhancing the reliability of the assembly sequence. The parameters for GATK (3.4-0-g7e26428) were set as follows: -cluster 2 -window 5 -stand_call_conf 50 -stand_emit_conf 10.0 -dcov 200 MQ0 >= 4.

#### 2.3.4. Bioinformatics Analysis of the Genome Sequence

Gene prediction was carried out on *Rhodococcus* sp. strain DN22 genome assembly utilizing glimmer3 with hidden Markov models. In addition, tRNAscan-SE [28], RNAmmer, and the Rfam database were utilized to identify tRNA, rRNA, and sRNA. The annotation of tandem repeats was acquired by Tandem Repeat Finder. Minisatellite DNA and microsatellite DNA selection was based on the quantity and length of repeat units. The Genomic Island Suite of Tools, with IslandPath-DIOMB, SIGI-HMM, and IslandPicker methods, was utilized for genomic island analysis. Prophage region prediction was conducted utilizing the PHAge Search Tool web server. CRISPRFinder was employed for the recognition of CRISPR.

In order to abstract the best hit for functional annotation, the blast alignment tool was utilized. The Kyoto Encyclopedia of Genes and Genomes (KEGG), Clusters of Orthologous Groups (COG), Non-Redundant Protein Database, Swiss-Prot, Gene Ontology (GO) [29], Translation of EMBL, and Evolutionary Genealogy of Genes: Non-supervised Orthologous Groups were applied with the aim of general function annotation. Additionally, the virulence factor and resistance gene identification were conducted using the core dataset in Virulence Factors of Bacterial Pathogens (Virulence Factor Database) and the Antibiotic Resistance Genes Database. Enzymes related to carbohydrates were identified based on Carbohydrate-Active enZYmes (CAZy). EffectiveT3 was utilized to detect type III secretion system effector proteins.

### 2.4. Proteomics and Metabolomics

#### 2.4.1. Bacterial Cell Pellet Preparation

Based on previous experiments on bacterial growth and RDX degradation detection, the weight of bacterial cells before RDX was completely degraded was too low for proteomics and metabolomics research. Therefore, more bacterial cells were prepared for RDX degradation detection, as well as for subsequent proteomics and metabolomics. Initially, *Rhodococcus* sp. strain DN22 was cultured in LB medium until the early stationary phase. Afterwards, bacteria were collected through centrifugation (4500 r/min, 10 min; 9000× *g*, 10 min), and washed twice in PBS (9000× *g*, 10 min). Approximately 300 mg of bacterial cells in PBS were then inoculated into the medium utilizing RDX as an exclusive nitrogen source.

First, RDX degradation by more bacterial cells was detected. The experiment was performed in triplicate with the RDX-free medium as the control. RDX levels were detected every 2 h by HPLC, to provide a reference for the harvesting time of the bacterial cells for proteomics and metabolomics.

Subsequently, bacterial cell pellets for proteomics and metabolomics were prepared. The experiment was performed in quintuplicate with the RDX-free medium as the control. After 7 h, the bacteria were harvested through centrifugation (4000 r/min, 10 min; 9000× *g*, 10 min), and washed twice in Wahaha purified water (9000× *g*, 10 min). Each bacterial sample from an Erlenmeyer flask was divided into two parts for proteomics and metabolomics analysis. The cells were frozen swiftly with liquid N_2_ and subsequently kept at a temperature of −80 °C. In addition, RDX levels at 0 h and 7 h were detected by HPLC.

#### 2.4.2. Protein Extraction, Digestion, and LC-MS/MS Analysis

The bacteria were resuspended in SDT lysate consisting of 4% (*w*/*v*) SDS, 100 mmol/L Tris/HCl (pH 7.6), and 0.1 mmol/L DTT, and disrupted through sonication at a low temperature. After an 8 min boiling water bath, every sample was centrifuged at 12,000× *g* for 20 min, with the supernatant being reserved. Bicinchoninic acid assay was utilized for protein concentration measurement. Samples were digested using trypsin through a filter-aided proteome preparation method [30]. After being dried by a vacuum centrifuge concentrator, peptides were dissolved in 0.1% HCOOH. Peptide concentration was measured at OD_280_ using Multiskan FC (Thermo Scientific, Waltham, MA, USA). LC-MS/MS analysis was conducted utilizing 400 ng peptides for each sample.

The peptide mixture was separated by utilizing the Easy-nLC system, with solution A containing 0.1% HCOOH and H_2_O, and solution B containing 0.1% HCOOH, 84% CH_3_CN, and H_2_O. 95% of solution A was utilized for chromatographic column equilibration. The samples were loaded onto a loading column (Thermo Scientific Acclaim PepMap100, 100 μm × 2 cm, nanoViper C_18_) by an autosampler and then separated through using an analytical column (Thermo Scientific EASY column, 25 cm, ID75 μm, 1.9 μm) with a flow rate of 300 nL/min. A 90 min gradient was applied, with solution B starting at 0% at 0 min, increasing to 7% at 3 min, 30% at 68 min, 55% at 83 min, and reaching 100% at 85 min, until 90 min.

A Q-Exactive HFX mass spectrometer was utilized to perform label-free quantification analysis for each sample. Positive ion detection was employed as the detection method, with a scan range of 300–1800 *m*/*z* for the parent ion. The resolution of the MS1 was 60,000 at 200 *m*/*z*, whilst the automatic gain control target was 1 × 10^6^. The maximum IT was 50 ms. The dynamic exclusion time was 30.0 s. The mass/charge ratios of the peptides and peptide fragments were collected as follows: after every full scan, 20 fragment graphs (MS2 scan) were collected, utilizing HCD as the MS2 activation type; the isolation window was set at 2 *m*/*z*, and the resolution of the MS2 was 15,000 at 200 *m*/*z*; the normalized collision energy was 27 eV, and underfill was 0.1 %.

#### 2.4.3. Data Processing and Bioinformatics Analysis of Proteomics

MaxQuant was utilized to process the RAW mass spectrometry files against the database consisting of *Rhodococcus erythropolis* DN1 proteomes in UniProt (UP000016045, standard) and *Rhodococcus* sp. DN22 proteins in UniProt (Q09Q55 and Q2PZX0). The parameters were set as follows: carbamidomethyl (C) as the fixed modification; oxidation (M) and acetyl (protein N-term) as variable modifications; trypsin/P enzyme specific; a maximum of 2 missed cleavages; Orbitrap as instrument type; 20 ppm first search peptide tolerance; and 4.5 ppm main search peptide tolerance. In addition, the option of match between runs was enabled with a 0.4 min match time window and a 20 min alignment time window.

The mass spectrometry proteomics data have been deposited to the ProteomeXchange Consortium via the PRIDE [31] partner repository with the dataset identifier PXD044565.

The results (proteinGroups) obtained from MaxQuant were imported into Perseus to conduct statistical analysis. The transform process was executed with default values. The rows were filtered based on categorical column, encompassing only identified by site, reverse, as well as potential contaminant. The rows were further filtered based on valid values with min = 10. Principal component analysis was carried out. The heatmap was plotted with the distance set as the Pearson correlation after the Z-score. After categorical annotation rows and analysis of the volcano plot (S0 = 1, FDR = 0.05), the datasheet for subsequent analysis was acquired. The significantly differentially expressed proteins were defined as *p*-value < 0.05, and difference (log_2_(foldchange)) < log_2_(2/3) or > log_2_(3/2), calculated as the RDX group/RDX-free group by five biological replicates. A volcano plot was ultimately obtained based on these thresholds.

The significantly differentially expressed proteins were used for bioinformatics analysis. The Subcellular Localization Predictive System (CELLO) [32] was utilized to analyze their subcellular localization. Additionally, Interproscan [33] was utilized for structural domain prediction. Enrichment analysis was accomplished utilizing Fisher’s exact test to uncover the structural domain enrichment characteristics of the significantly differentially expressed proteins.

Gene ontology functional annotation of the significantly differentially expressed proteins (upregulated and downregulated proteins individually) was executed using Blast2GO (v4.5 pipeline 5) [34] software with the biological process, molecular function, and cellular component [29] included. Meanwhile, the protein count was quantified at the GO secondary functional annotation level.

The proteins were analyzed and annotated through KEGG pathway database [35]. The numerous metabolic pathways and the pathway attribution relationships were represented graphically, enabling a more accessible observation of metabolic pathways that the significantly differentially expressed proteins participated in. To reveal the overall metabolic pathway enrichment characteristics, Fisher’s exact test was utilized in the KEGG pathway enrichment analysis of the significantly differentially expressed proteins. The significantly differentially expressed proteins and the proteins that corresponded to the points in the volcano plot were compared according to the KEGG annotation results, with the difference significance obtained utilizing Fisher’s exact test, so as to identify the enriched pathway category of the significantly differentially expressed proteins (upregulated and downregulated proteins individually).

#### 2.4.4. Metabolite Extraction and UHPLC-Q-TOF MS

Approximately 80 mg of bacterial cells for each sample were weighed and reserved. An amount of 1 mL of extracting solution (precooled at 4 °C) comprising CH_3_OH, CH_3_CN and H_2_O in a ratio of 2:2:1 (*v*/*v*) was added and vortexed. Every sample was disrupted by sonication at low temperature for a duration of 30 min. After annealing at a temperature of −20 °C for 10 min, the samples were centrifuged (14,000× *g*, 20 min, 4 °C) and the supernatant was then reserved. Finally, 2 μL of the metabolite mixture was utilized for UHPLC-Q-TOF MS. The samples were consecutively analyzed in random order to avoid the effect of instrument detection signal fluctuation. In addition, to monitor and assess system stability and experimental result reliability, the samples were equally mixed to prepare quality control samples, which were tested before, during, and after UHPLC-Q-TOF MS analysis.

Analysis was conducted utilizing a UHPLC system (1290 Infinity LC, Agilent, Stevens Creek Blvd, Santa Clara, CA, USA) coupled to a quadrupole time-of-flight mass spectrometer (AB Sciex TripleTOF 6600, 500 Old Connecticut Path, Framingham, MA, USA). More precisely, a 2.1 mm × 100 mm ACQUIY UPLC BEH Amide 1.7 µm column (Waters, IDA Business Park, Drinagh, Wexford Y35 D431, Ireland) was utilized for hydrophilic interaction liquid chromatography separation. In the positive and negative modes of electrospray ionization, the mobile phase consisted of solution A (25 mmol/L CH_3_COONH_4_ and 25 mmol/L NH_3_·H_2_O in H_2_O) and solution B (CH_3_CN). Solution B was initially 95% for 0.5 min, linearly decreased to 65% in 6.5 min, subsequently decreased to 40% in 1 min, maintained for 1 min, and increased to 95% within 0.1 min, with a 3 min re-equilibration period applied.

The electrospray ionization source conditions were configured as follows: ion source Gas1 and ion source Gas2 set at 60, curtain gas (CUR) set at 30, source temperature set at 600 °C, and IonSpray Voltage Floating (ISVF) set at ±5500 V. During MS-only acquisition, the instrument was programmed to capture the *m*/*z* range of 60–1000 Da, with the accumulation time for the TOF MS scan set at 0.20 s/spectrum. During auto MS/MS acquisition, the detection range of *m*/*z* was 25–1000 Da, with the accumulation time for the product ion scan being 0.05 s/spectrum. The product ion scan was obtained utilizing information-dependent acquisition with the high-sensitivity mode selected. The parameters were set as follows: the collision energy was set at a fixed value of 35 V (±15 eV), the declustering potential was set as 60 V (+) and −60 V (−), and isotopes within 4 Da were excluded, 10 candidate ions monitored each cycle.

#### 2.4.5. Data Processing and Bioinformatics Analysis of Metabolomics

The mass spectrometry data underwent conversion to MzXML files utilizing ProteoWizard MSConvert prior to being imported into XCMS, for peak alignment, retention time correction, as well as peak area extraction. The extracted data went through identification of metabolite structure, data preprocessing, evaluation of experimental data quality, and ultimately data analysis.

Searching was conducted utilizing an in-house database in Shanghai Applied Protein Technology [36,37]. The metabolites were structurally identified through matching the retention time, molecular mass (with a 25 ppm molecular mass error tolerance), secondary fragmentation spectra, as well as collision energy of the metabolites present in the database. Additionally, manual secondary checking was carried out for rigorous checks and confirmation of the identification results. The metabolites were identified at Level 2 or higher [38].

All of the metabolites detected in positive and negative ion modes, unidentified ones included, were utilized for differential analysis on the basis of univariate analysis. Differential metabolites with a *p*-value < 0.05 and fold change < 0.67 or >1.5 were visualized using volcano plots. Orthogonal partial least squares discriminant analysis (OPLS-DA) can remove noise unconcerned with categorical information. An OPLS-DA score plot includes predictive and orthogonal principal components. Variable importance for the projection (VIP) acquired from the OPLS-DA model serves as an essential tool for measuring the effect strength and explanatory power of every metabolite’s expression pattern on every group’s categorical discrimination, excavating differential metabolite molecules that have biological significance.

The significantly differential metabolites were defined as *p*-value < 0.05 and OPLS-DA VIP > 1, with qualitative names. The significantly differential metabolites in positive and negative ion modes were merged before KEGG pathway annotation and analysis. The KEGG pathway enrichment analysis utilized Fisher’s exact test to analyze and quantify the metabolite enrichment significance level in every pathway. Utilizing a differential abundance score [39], which is a pathway-based analysis method of metabolic alterations, the overall or average changes in all metabolites in one pathway can be attained. The differential abundance score was calculated for each metabolic pathway that was significantly enriched.

#### 2.4.6. Combined Analysis of Proteomics and Metabolomics

For the significantly differentially expressed proteins and significantly differential metabolites defined as previously mentioned, the KEGG pathway annotation information was combined and shown in KEGG pathway maps, with upregulated proteins/metabolites in red and downregulated ones in green. In addition, based on the KEGG pathway enrichment analysis of significantly differentially expressed proteins and significantly differential metabolites, the KEGG pathways that were significantly enriched (*p*-value < 0.05) in both proteomics and metabolomics were focused.

## 3. Results

### 3.1. Growth Curve of Rhodococcus sp. Strain DN22 in LB Medium

The growth curve of *Rhodococcus* sp. Strain DN22 in the LB medium was detected (Figure S1A). The timeframe from 0 to 80 h was segmented into three phases: lag phase (0–20 h), logarithmic phase (20–46 h), and stationary phase (46–80 h). It was determined that OD_530_ was higher than OD_600_ under this condition, suggesting that OD_530_ may be preferable for the detection of *Rhodococcus* sp. Strain DN22 growth. In addition, the early stationary phase was determined (46–60 h), in which the bacteria were employed for subsequent experiments in the medium utilizing RDX as an exclusive nitrogen source.

### 3.2. Growth Curve of Rhodococcus sp. Strain DN22 and RDX Degradation by Rhodococcus sp. Strain DN22 in the Medium Utilizing RDX as an Exclusive Nitrogen Source

The growth curve of *Rhodococcus* sp. Strain DN22 in the medium utilizing RDX as an exclusive nitrogen source was detected, with the RDX-free medium as the control. Meanwhile, RDX degradation was analyzed by HPLC. The results are shown in Figure S1B. RDX was undetectable within 34 h, and bacterial growth continued up to 100 h. OD_530_ was higher than OD_600_ under this condition, consistent with previous results.

### 3.3. Complete Genome Sequencing Analysis

The total length of the complete genome sequence of *Rhodococcus* sp. Strain DN22 is 7,435,512 bp, including a circular chromosome (6,509,401 bp) and six plasmids (three circular plasmids (plasmid1, 323,798 bp; plasmid4, 128,752 bp; plasmid5, 38,509 bp) and three linear plasmids (plasmid2, 231,447 bp; plasmid3, 175,620 bp; plasmid6, 27,985 bp)). In total, 7203 genes are present in *Rhodococcus* sp. strain DN22, with a 62.29% GC content. The fasta sequence file for the genome of *Rhodococcus* sp. strain DN22 is shown in Table S1. Sequencing data statistics for DNBSEQ platform are displayed in Table 1. Reads data statistics for PacBio Sequel II platform are displayed in Table 2.

**Figure 1:**
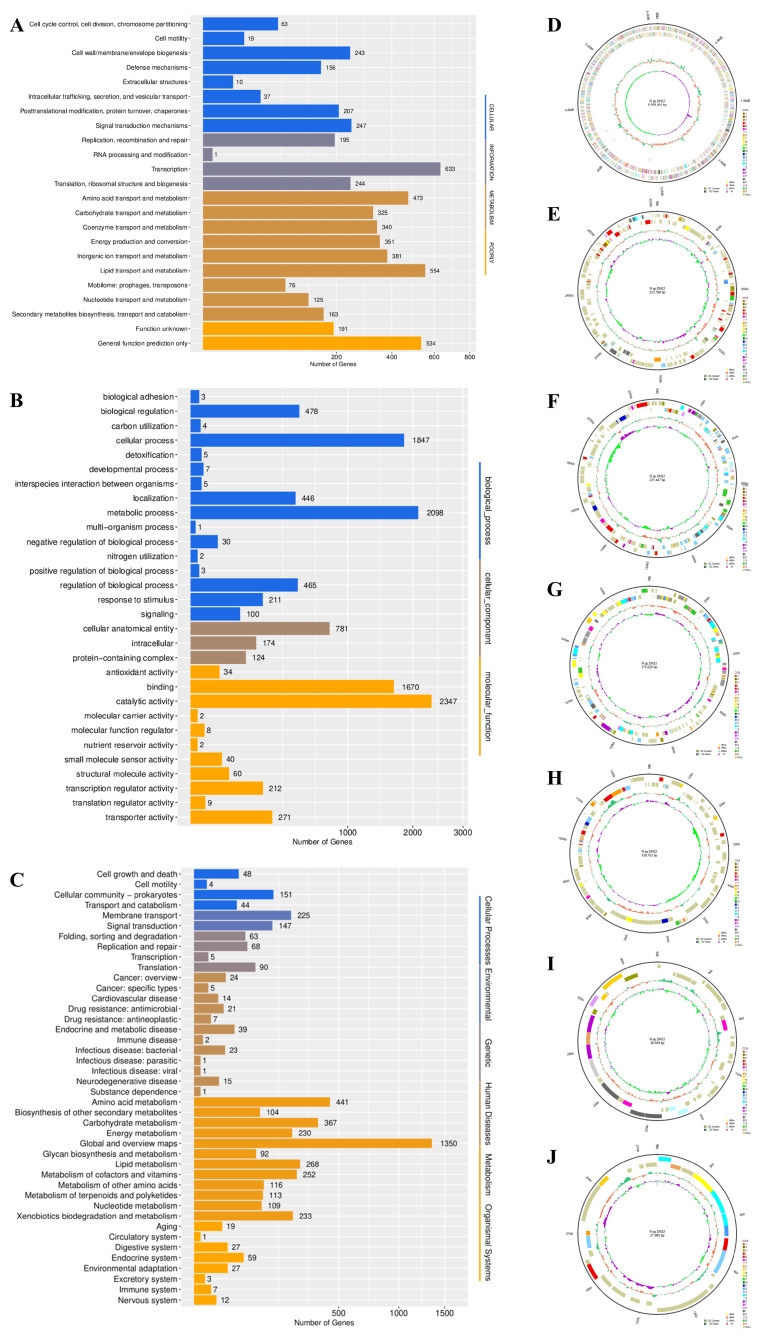
Complete genome sequencing analysis. (**A**) COG functional classification plot. (**B**) GO functional classification plot. (**C**) KEGG pathway classification plot. (**D**) Chromosome circle graph. A high-resolution figure is displayed in Figure S2. (**E**–**J**) Plasmid circle graphs for plasmid1–6. High-resolution figures are displayed in Figures S3–S8.

The COG functional classification plot is displayed in Figure 1A, indicating the likely involvement of numerous genes in signal transduction mechanisms, transcription, lipid transport, and metabolism, among others. The GO functional classification plot is displayed in Figure 1B, revealing that various genes were connected with the metabolic process, cellular anatomical entity, and catalytic activity, among others. The KEGG pathway classification plot is displayed in Figure 1C, suggesting that some genes were potentially in connection with cellular community-prokaryotes, membrane transport, translation, and amino acid metabolism, among others. Notably, 233 genes were observed to be involved in xenobiotics biodegradation and the metabolism pathway.

The *Rhodococcus* sp. strain DN22 genome was presented utilizing Circos software (v 0.69-8, 15 Jun 2019, Perl 5.032001), comprehensively showcasing genes, ncRNA, repetitive sequences, annotation information, methylation, GC content, and GC skew. A chromosome circle graph is displayed in Figure 1D, while plasmid circle graphs are displayed in Figure 1E–J, representing plasmid1–6, respectively.

### 3.4. Proteomic and Metabolomic Analysis

#### 3.4.1. RDX Degradation by *Rhodococcus* sp. Strain DN22 Cells in the Medium Utilizing RDX as an Exclusive Nitrogen Source

**Figure 2:**
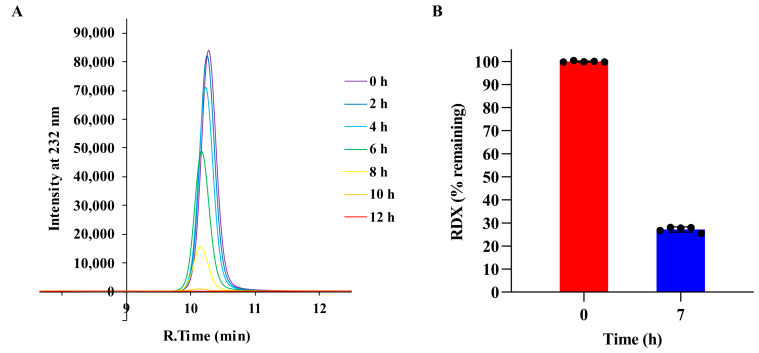
Detection of RDX degradation by 300 mg of *Rhodococcus* sp. strain DN22 cells. (**A**) The partial chromatogram (one of the three samples) showing the chromatographic peak of RDX, with different colors representing different time points. (**B**) RDX levels at 0 h and 7 h in the medium utilizing RDX as an exclusive nitrogen source, in which condition RDX was degraded by 300 mg of *Rhodococcus* sp. strain DN22 cells, to prepare bacterial cell pellets for proteomics and metabolomics. RDX levels are shown by the filled circles (n = 5).

Approximately 300 mg of *Rhodococcus* sp. strain DN22 was inoculated into the medium utilizing RDX as an exclusive nitrogen source, using the RDX-free medium as the control. RDX levels were detected every 2 h by HPLC, and the results (in triplicate) are shown in Figure S9A. RDX was not detectable within 12 h. The partial chromatogram (one of the three samples) showing the chromatographic peak of RDX is displayed in Figure 2A, with different colors representing different time points, illustrating the RDX degradation process by *Rhodococcus* sp. strain DN22.

Bacteria were harvested at 7 h to prepare bacterial cell pellets for proteomics and metabolomics. At that time, approximately 27% of the RDX remained (Figure 2B).

#### 3.4.2. Proteomic Analysis

**Figure 3:**
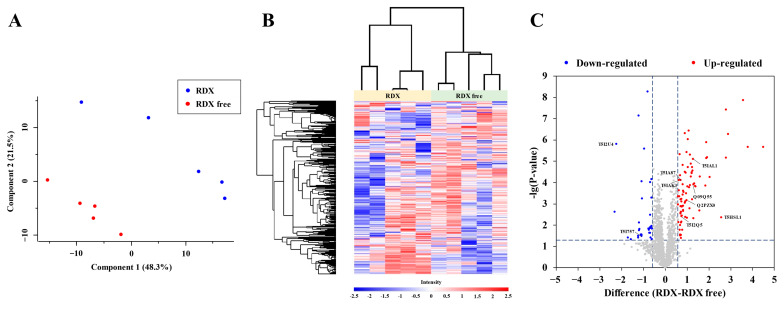
Proteomic quantification results. (**A**) Principal component analysis of the two groups, showing components 1 and 2. The red dots indicate the RDX-free group, while the blue dots indicate the RDX group. (**B**) Heatmap of the two groups, displaying the differences between them, suggesting a reliable quantification. (**C**) Volcano plot of the two groups, with upregulated proteins in red and downregulated proteins in blue.

Label-free quantification was carried out on the RDX group (in the medium utilizing RDX as an exclusive nitrogen source) and the RDX-free group (the control, in the RDX-free medium). A total of 3186 proteins were identified between the two groups through MaxQuant. The results of proteomics data are displayed in Table S2. In total, 2502 proteins were reserved after filtering the rows. To acquire a general overview of data quality, principal component analysis was performed. As displayed in Figure 3A, the proteins of every group clustered together and were distinct from the other group. Furthermore, the heatmap revealed that the differences in protein expression between the two groups were significant (Figure 3B). A volcano plot for the RDX and RDX-free groups is displayed in Figure 3C, with upregulated proteins in red and downregulated proteins in blue. A total of 83 proteins were upregulated and 32 proteins were downregulated in the RDX group, in comparison with the RDX-free group. Some significantly differentially expressed proteins are noteworthy and will be discussed later.

**Figure 4:**
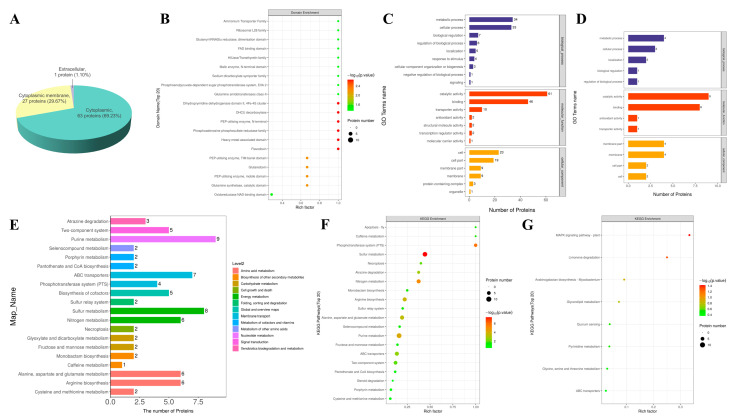
Functional analysis of the significantly differentially expressed proteins. (**A**) Subcellular localization analysis of the significantly differentially expressed proteins. (**B**) Structural domain enrichment analysis of the significantly differentially expressed proteins. (**C**) Statistical analysis of GO annotation (level 2) of the upregulated proteins. (**D**) Statistical analysis of GO annotation (level 2) of the downregulated proteins. (**E**) KEGG pathway annotation and attribution histogram of the significantly differentially expressed proteins. (**F**) KEGG pathway enrichment bubble diagram of the upregulated proteins. (**G**) KEGG pathway enrichment bubble diagram of the downregulated proteins.

Significantly differentially expressed proteins were utilized for the functional analysis. The subcellular localization analysis is displayed in Figure 4A. The pie chart shows the protein distribution across organelles (cytoplasmic, 63 proteins; cytoplasmic membrane, 27 proteins; extracellular, 1 protein). Figure 4B demonstrates the structural domain enrichment analysis of the significantly differentially expressed proteins. Dihydroprymidine dehydrogenase domain II, 4Fe-4S cluster, OHCU decarboxylase, PEP-utilising enzyme, N-terminal, phosphoadenosine phosphosulfate reductase family, heavy-metal-associated domain, and flavodoxin were distinctly enriched.

The statistical analysis of gene ontology annotation (level 2) is presented in Figure 4C (upregulated proteins) and Figure 4D (downregulated proteins). For the upregulated proteins, the enriched biological processes were metabolic process, cellular process, biological regulation, regulation of biological process, localization, response to stimulus, cellular component organization or biogenesis, negative regulation of biological process, and signaling; the enriched molecular functions were catalytic activity, binding, transporter activity, antioxidant activity, structural molecule activity, transcription regulator activity, and molecular carrier activity; and the enriched cellular components were cell, cell part, membrane part, membrane, protein-containing complex, and organelle. For the downregulated proteins, the enriched biological processes were metabolic process, cellular process, localization, biological regulation, and regulation of biological process; the enriched molecular functions were catalytic activity, binding, antioxidant activity, and transporter activity; and the enriched cellular components were membrane part, membrane, cell part, and cell. The results revealed that the existence or lack of RDX had major impacts on cells in essential aspects, including cellular process, biological regulation, catalytic activity, transporter activity, cell, and membrane.

Figure 4E displays the KEGG pathway annotation and attribution histogram for the significantly differentially expressed proteins. The attribution of the metabolic pathways where more significantly differentially expressed proteins participated included nucleotide metabolism, membrane transport, energy metabolism, and amino acid metabolism. The findings revealed that the existence or lack of RDX would affect cells in substance transport and fundamental metabolic processes. Figure 4F,G display KEGG pathway enrichment bubble diagrams of upregulated and downregulated proteins, respectively. For the upregulated proteins, the enriched KEGG pathways (top 20) were apoptosis-fly, caffeine metabolism, phosphotransferase system (PTS), sulfur metabolism, necroptosis, atrazine degradation, nitrogen metabolism, monobactam biosynthesis, arginine biosynthesis, sulfur relay system, alanine, aspartate and glutamate metabolism, selenocompound metabolism, purine metabolism, fructose and mannose metabolism, ABC transporters, two-component system, pantothenate and CoA biosynthesis, steroid degradation, porphyrin metabolism, and cysteine and methionine metabolism. Among these, nitrogen metabolism and two-component system were likely to be the effects of the existence of RDX in the medium. For the downregulated proteins, the enriched KEGG pathways were MAPK signaling pathway-plant, limonene degradation, arabinogalactan biosynthesis-*Mycobacterium*, glycerolipid metabolism, quorum sensing, pyrimidine metabolism, glycine, serine and threonine metabolism, and ABC transporters. The results indicated that the existence or lack of RDX could affect the biosynthesis and metabolism of amino acids, which were of great importance in bacterial survival and growth, signifying that the nitrogen in the RDX molecules might participate in the central processes of the bacteria.

#### 3.4.3. Metabolomic Analysis

**Figure 5:**
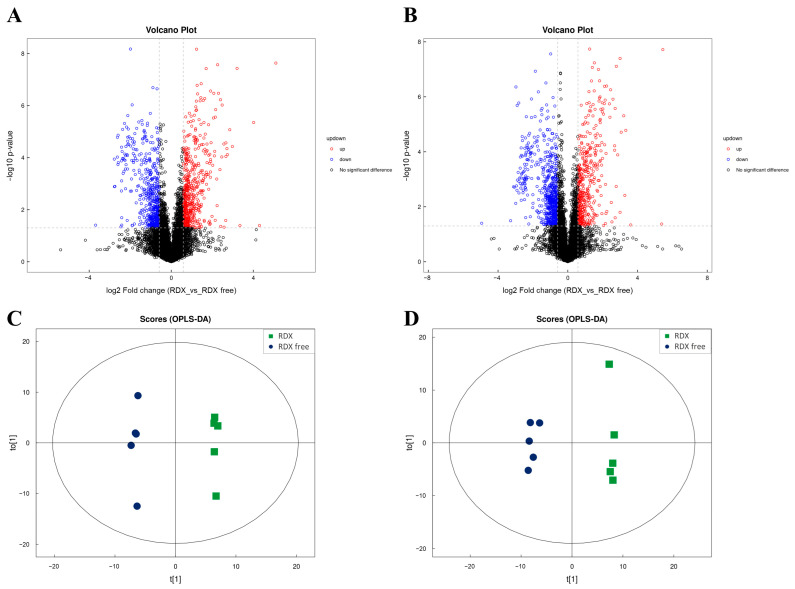
Metabolomic univariate and multidimensional statistical analysis. (**A**) Positive ion mode volcano plot for the two groups. (**B**) Negative ion mode volcano plot for the two groups. (**C**) Positive ion mode OPLS-DA score plot of the two groups. (**D**) Negative ion mode OPLS-DA score plot of the two groups.

A total of 641 metabolites were identified by positive ion mode and 415 metabolites were identified by negative ion mode, resulting in a total of 1056 after merging. Table S3 presents the qualitative and quantitative results of metabolites. The positive and negative ion mode volcano plots yielded by univariate statistical analysis are presented in Figure 5A,B, respectively. Based on multidimensional statistical analysis, positive and negative ion mode OPLS-DA score plots were generated, as displayed in Figure 5C,D. The results demonstrated that the OPLS-DA model could differentiate between the two groups.

There were 130 significantly differential metabolites determined, 72 by positive ion mode and 58 by negative ion mode, among which 78 were upregulated and 52 were downregulated in the RDX group in comparison with the RDX-free group. Most of the significantly differential metabolites were organic acids and derivatives, organic oxygen compounds, lipids, and lipid-like molecules. The findings suggested that metabolic processes related to these substances could be affected by the existence or lack of RDX in the medium. In addition, some significantly differential metabolites were found to be nucleosides, nucleotides, and analogues, which might be involved in central bacterial processes. Furthermore, some organoheterocyclic compounds and benzenoids were observed to be significantly differential metabolites.

Notably, NADH (β-nicotinamide adenine dinucleotide, reduced) was upregulated in the RDX group. NAD(P)H, serving as an electron donor, was consumed during RDX degradation [12,13]. Therefore, it was supposed that NADH production was facilitated abundantly in the RDX group for the redox reaction process in RDX degradation.

**Figure 6:**
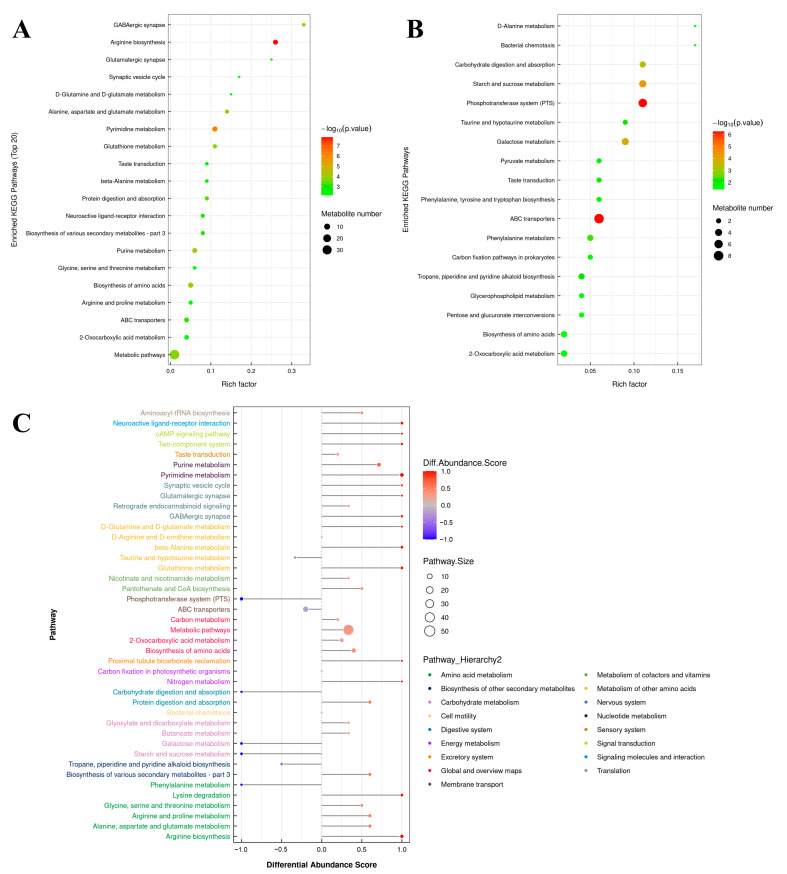
Functional analysis of the significantly differential metabolites. (**A**) The KEGG pathway enrichment bubble plot for upregulated metabolites. (**B**) The KEGG pathway enrichment bubble plot for downregulated metabolites. (**C**) Differential abundance score plot for significantly enriched metabolic pathways (classification and attribution according to Pathway_Hierarchy2).

The results of the metabolic pathway enrichment analysis are depicted as bubble plots, with Figure 6A for upregulated metabolites and Figure 6B for downregulated metabolites. For the upregulated metabolites, enriched KEGG pathways (top 20) were GABAergic synapse, arginine biosynthesis, glutamatergic synapse, synaptic vesicle cycle, D-glutamine and D-glutamate metabolism, alanine, aspartate and glutamate metabolism, pyrimidine metabolism, pyrimidine metabolism, taste transduction, beta-alanine metabolism, beta-alanine metabolism, neuroactive ligand-receptor interaction, biosynthesis of various secondary metabolites-part 3, purine metabolism, glycine, serine and threonine metabolism, biosynthesis of amino acids, arginine and proline metabolism, ABC transporters, 2-oxocarboxylic acid metabolism, and metabolic pathways. For the downregulated metabolites, enriched KEGG pathways were D-alanine metabolism, bacterial chemotaxis, carbohydrate digestion and absorption, starch and sucrose metabolism, phosphotransferase system (PTS), taurine and hypotaurine metabolism, galactose metabolism, pyruvate metabolism, taste transduction, phenylalanine, tyrosine and tryptophan biosynthesis, ABC transporters, phenylalanine metabolism, carbon fixation pathways in prokaryotes, tropane, piperidine and pyridine alkaloid biosynthesis, glycerophospholipid metabolism, pentose and glucuronate interconversions, biosynthesis of amino acids, and 2-oxocarboxylic acid metabolism. A differential abundance score plot for significantly enriched metabolic pathways, classified and attributed according to Pathway_Hierarchy2, is displayed in Figure 6C. Pathways hierarchically attributed to amino acid metabolism, global and overview maps, and nucleotide metabolism, were observed to be more inclined towards upregulation. On the other hand, pathways hierarchically attributed to membrane transport and carbohydrate metabolism were observed to be more inclined towards downregulation. It was noteworthy that nitrogen metabolism, which was hierarchically attributed to energy metabolism, displayed an inclination towards upregulation.

## 4. Discussion

### 4.1. Analysis of Some Significantly Differentially Expressed Proteins

Endoribonuclease L-PSP (T5I2U4), which could inhibit protein synthesis by cleavage of mRNA, was significantly upregulated in the RDX-free group, suggesting that cells might reduce energy expenditure by inhibiting protein synthesis when there was a lack of a nitrogen source. Moreover, the significant downregulation of secreted protein (T5I757) in the RDX group implied that the bacteria were likely to slacken biological processes in response to the lack of a nitrogen source in the medium.

Oxidoreductase (Q09Q55) and cytochrome P450 (Q2PZX0) [19,20], which participate in RDX degradation in *Rhodococcus* sp. strain DN22, were significantly upregulated in the RDX group and will be discussed in detail later. NAD(P)H is essential for RDX degradation [12]. Malate dehydrogenase (T5IA87), catalyzing the oxidation of malate to oxoacetate in the tricarboxylic acid cycle, in which step NADH was produced; and alcohol dehydrogenase (T5IAL1), oxidizing ethanol and reducing NAD^+^ to NADH, were significantly upregulated in the RDX group, suggesting that bacteria actively facilitated the degradation of RDX. Additionally, the accumulation of NH_3_ resulting from RDX biodegradation [9] might have contributed to the significant upregulation of glutamine synthetase (T5I2Q5), which facilitates the transformation of NH_4_^+^. In addition, the significant upregulation of cell division protein CrgA (T5IAK3) indicated that cells could coordinate reproductive growth when provided with a nitrogen source. Furthermore, RNA helicase (T5HSL1) was significantly upregulated, indicating that cells engaged in RNA metabolism under the condition of adequate nitrogen supply.

### 4.2. Combined Analysis of Proteomics and Complete Genome Sequencing: Focusing on the Two Proteins Related to RDX Degradation

In the data processing of proteomics above, *Rhodococcus* sp. DN22 proteins in UniProt (Q09Q55 and Q2PZX0) were incorporated into the database for MaxQuant searching. The results demonstrated that both proteins were detectable in the RDX and RDX-free groups and displayed significant upregulation in the RDX group. The phenomenon was in agreement with earlier studies on XplA (the protein responsible for RDX degradation) in *Rhodococcus rhodochrous* strain 11Y conducted by Dr. Neil C. Bruce [5,21].

Oxidoreductase (Q09Q55) and cytochrome P450 (Q2PZX0) were identified as fragment sequences on UniProt website. Sequence alignment for these two proteins with complete genome sequencing results in this study revealed that they were encoded by two genes in plasmid5 in *Rhodococcus* sp. strain DN22, verifying Dr. Nicholas Coleman’s previous conclusion [19]. The identity for oxidoreductase (Q09Q55) was 100%, while the identity for cytochrome P450 (Q2PZX0) was 99%, with one inconsistent amino acid, which will be analyzed later with proteomic data. *Rhodococcus rhodochrous* strain 11Y was another RDX degrading strain that has been thoroughly studied on the gene cluster involved in the RDX degradation [21]. Surprisingly, when performing sequence alignment for adrenodoxin reductase-like (Q8GPH8) and cytochrome P450-like protein XplA (Q8GPH7) from *Rhodococcus rhodochrous* strain 11Y with the entire sequences of the two genes (in plasmid5 in *Rhodococcus* sp. strain DN22) mentioned above, the identities were both 100%. These findings were in agreement with the conclusion of a previous investigation on conservation [20]. Table 3 displays the sequence alignment information.

Regarding the inconsistent amino acid mentioned above, the 232nd amino acid in cytochrome P450 (Q2PZX0) is glutamic acid (E), whereas it is aspartic acid (D) in plasmid5 from complete genome sequencing results in this study at the same position. In addition, it is aspartic acid (D) in cytochrome P450-like protein XplA (Q8GPH7) from *Rhodococcus rhodochrous* strain 11Y at the same position (the 235th amino acid). To analyze this inconsistent amino acid, pFind 3 software was utilized to process proteomic RAW mass spectrometry files against the database solely consisting of adrenodoxin reductase-like (Q8GPH8) and cytochrome P450-like protein XplA (Q8GPH7). The other parameters in pFind were at default values. It was found that the protein coverage of adrenodoxin reductase-like (Q8GPH8) reached 85.2%, and the protein coverage of cytochrome P450-like protein XplA (Q8GPH7) reached 95.1%, suggesting a high reliability. The pFind results indicated that it was aspartic acid (D) without any modification at that position. By utilizing of the open search algorithm, it was revealed that it was aspartic acid (D) exactly at that position. To summarize, the entire sequences of the two genes that encoded oxidoreductase and cytochrome P450 in *Rhodococcus* sp. strain DN22 were obtained through the complete genome sequencing results, and the sequences were validated through proteomic data.

The proteomic RAW mass spectrometry files were processed utilizing MaxQuant against the database solely consisting of adrenodoxin reductase-like (Q8GPH8) and cytochrome P450-like protein XplA (Q8GPH7). The other parameters were set according to the earlier method. As a result, both proteins were found to be upregulated in the RDX group, consistent with the earlier results of oxidoreductase (Q09Q55) and cytochrome P450 (Q2PZX0), making the matter more solid from another perspective.

In this study, the entire sequences of the two genes involved in RDX degradation in *Rhodococcus* sp. strain DN22 were acquired for the first time. In addition, advanced proteomics revealed that the critical proteins (oxidoreductase and cytochrome P450) that participated in RDX degradation in *Rhodococcus* sp. strain DN22 were upregulated in the RDX group.

### 4.3. Combined Analysis of Proteomics and Metabolomics

**Figure 7:**
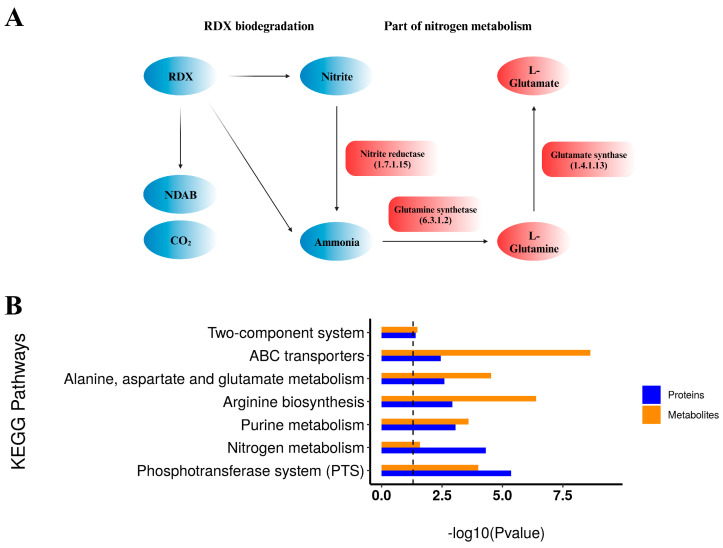
Combined analysis of proteomics and metabolomics. (**A**) Part of the KEGG map of nitrogen metabolism along with RDX biodegradation. Red bubbles represent upregulated proteins and metabolites in the RDX group in comparison with the RDX-free group. The illustration was created with BioRender.com. (**B**) Significantly enriched KEGG pathways (*p*-value < 0.05) for both differential proteomics and differential metabolomics.

Part of the KEGG map of nitrogen metabolism along with RDX biodegradation [9,12,13] is illustrated in Figure 7A, displaying upregulated proteins and metabolites in the RDX group as red bubbles, in comparison with the RDX-free group. In nitrogen metabolism, nitrite reductase was found to be significantly upregulated in the RDX group. Nitrite reductase can catalyze the reaction of nitrite into ammonia. Glutamine synthetase subsequently transforms ammonia into L-glutamine, which is further converted by glutamate synthase to L-glutamate. Both enzymes (glutamine synthetase and glutamate synthase) and their products (L-glutamine and L-glutamate) were significantly upregulated. It was previously reported that ammonia and nitrite (NO_2_^−^) were produced during RDX degradation [9]. Therefore, it was supposed that these significantly upregulated proteins and metabolites were due to the RDX degradation process.

Significantly enriched KEGG pathways (*p*-value < 0.05) for both differential proteomics and differential metabolomics are shown in Figure 7B, including two-component system, ABC transporters, alanine, aspartate and glutamate metabolism, arginine biosynthesis, purine metabolism, nitrogen metabolism, and phosphotransferase system (PTS). The two-component system can detect and convert environmental information, provoking adequate cellular responses [40]. The existence or lack of RDX in the medium appeared to contribute to the significant influence on the two-component system. ABC transporters and phosphotransferase system (PTS) were significantly enriched, indicating that the existence or lack of RDX could impact substance transport and carbohydrate uptake and metabolism. Alanine, aspartate and glutamate metabolism, arginine biosynthesis, and purine metabolism were significantly enriched, suggesting that these fundamental small molecules might be connected with the existence or lack of RDX. Additionally, nitrogen metabolism was also significantly regulated and discussed above.

## 5. Conclusions

Multi-omics strategies were utilized to investigate the biodegradation of RDX in *Rhodococcus* sp. strain DN22. The complete genome was sequenced and analyzed, and the entire sequences of genes that encoded the two proteins participating in RDX degradation were obtained, and were validated through proteomic data. Proteomics and metabolomics research with the existence or lack of RDX was performed. A total of 3186 proteins were identified between the two groups, with 115 proteins being significantly differentially expressed proteins. There were 1056 metabolites identified in total, among which 130 metabolites were significantly different. Through the combined analysis of differential proteomics and metabolomics, KEGG pathways including two-component system, ABC transporters, alanine, aspartate and glutamate metabolism, arginine biosynthesis, purine metabolism, nitrogen metabolism, and phosphotransferase system (PTS), were observed to be significantly enriched. This study is anticipated to expand the knowledge of *Rhodococcus* sp. strain DN22, as well as advancing the understanding of microbial degradation.

## Figures and Tables

**Table 1:** Sequencing data statistics for the DNBSEQ platform.

Insert Size (bp)	Reads Length (bp)	Raw Data (Mb)	Adapter (%)	Duplication (%)	Total Reads (#)	Filtered Reads (%)	Low Quality Filtered Reads (%)	Clean Data (Mb)
350	(150:150)	1169	1.08	0.23	7,798,630	2.23	0	1143

**Table 2:** Reads data statistics for the PacBio Sequel II platform.

Valid ZWM Number (#)	Subreads Number (#)	Subreads Total Bases (bp)	Subreads Mean Length (bp)	Subreads N50 (bp)	Subreads N90 (bp)	Subreads Max Length (bp)	Subreads Min Length (bp)
83,467	710,098	6,965,000,535	9808	10,364	6752	195,292	1000

**Table 3:** Sequence alignment information.

Inputs of Sequence Alignment (Sequences from UniProt (Left) with Complete Genome Sequencing Results in This Study (Right))		Identities
Oxidoreductase (Q09Q55)	Nucleotide sequence of plasmid #5	150/150 (100%)
Cytochrome P450 (Q2PZX0)	Nucleotide sequence of plasmid #5	235/236 (99%)
Adrenodoxin reductase-like (Q8GPH8)	The entire sequence of the gene encoding oxidoreductase in plasmid #5	425/425 (100%)
Cytochrome P450-like protein XplA (Q8GPH7)	The entire sequence of the gene encoding cytochrome P450 in plasmid #5	552/552 (100%)

## Data Availability

Complete genome of *Rhodococcus* sp. DN22 is available on NCBI (Accession: CP133326-CP133332). The mass spectrometry proteomics data have been deposited to the ProteomeXchange Consortium via the PRIDE partner repository with the dataset identifier PXD044565.

## Supplementary Materials

The following supporting information can be downloaded at: https://www.mdpi.com/article/10.3390/microorganisms12010076/s1, Figure S1: The growth curve of *Rhodococcus* sp. strain DN22. Figure S2: A high-resolution figure for Figure 1D (Chromosome circle graph). Figure S3–S8: High-resolution figures for Figure 1E–J (Plasmid circle graphs for plasmid1–6). Figure S9: RDX degradation by 300 mg of *Rhodococcus* sp. strain DN22 cells in the medium utilizing RDX as an exclusive nitrogen source. Table S1: The fasta sequence file for the genome of *Rhodococcus* sp. strain DN22. Table S2: The results of proteomics data. Table S3: The qualitative and quantitative results of metabolites.
