# Supplementary material for: Multi-Omics Strategies to Investigate the Biodegradation of Hexahydro-1,3,5-trinitro-1,3,5-triazine in Rhodococcus sp. Strain DN22
Source: Microorganisms. 2023 Dec 30;12(1):76. doi: 10.3390/microorganisms12010076 (PMC10820124; doi:10.3390/microorganisms12010076)
Supplement: Supplementary file 1 [file microorganisms-12-00076-s001.zip › Supplementary Materials.pdf]

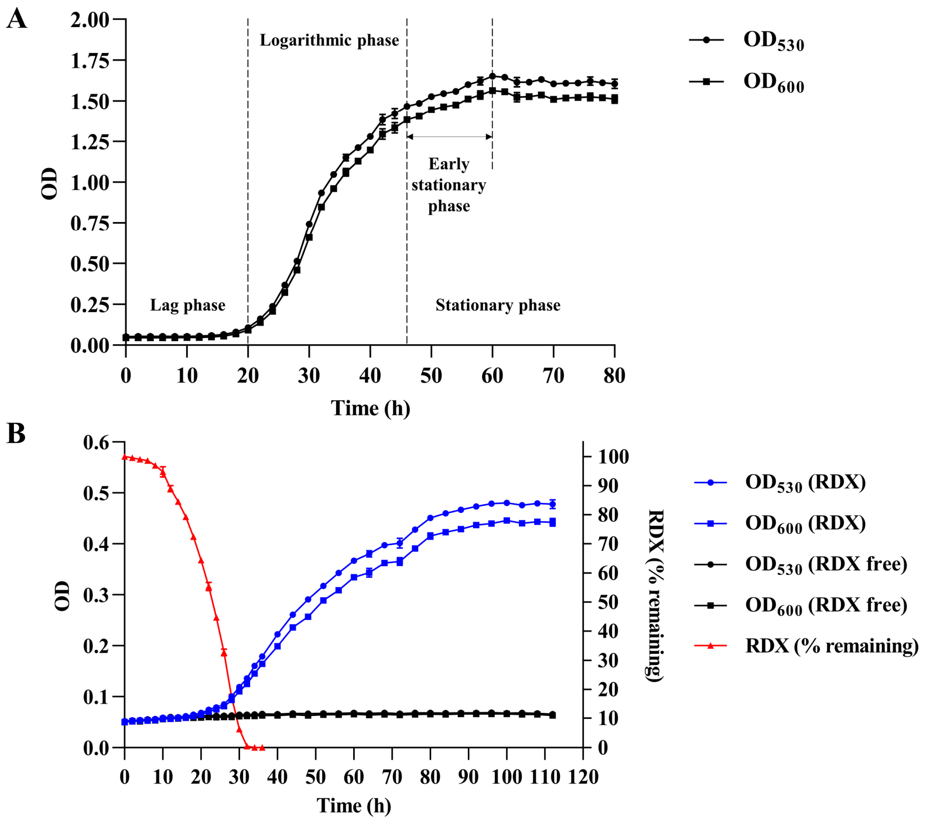

Figure S1. The growth curves of *Rhodococcus* sp. strain DN22.

(A) The growth curve of *Rhodococcus* sp. strain DN22 in LB medium. OD_{530} is shown in filled circles, and OD_{600} in filled squares (n = 3). **(B)** The growth curve of *Rhodococcus* sp. strain DN22 in the medium utilizing RDX as an exclusive nitrogen source (OD_{530} shown in filled blue circles, and OD_{600} in filled blue squares), with RDX-free medium as the control (OD_{530} shown in filled black circles, and OD_{600} in filled black squares) (n = 3). RDX levels are shown in filled red triangles (right Y axis) (n = 3).

A

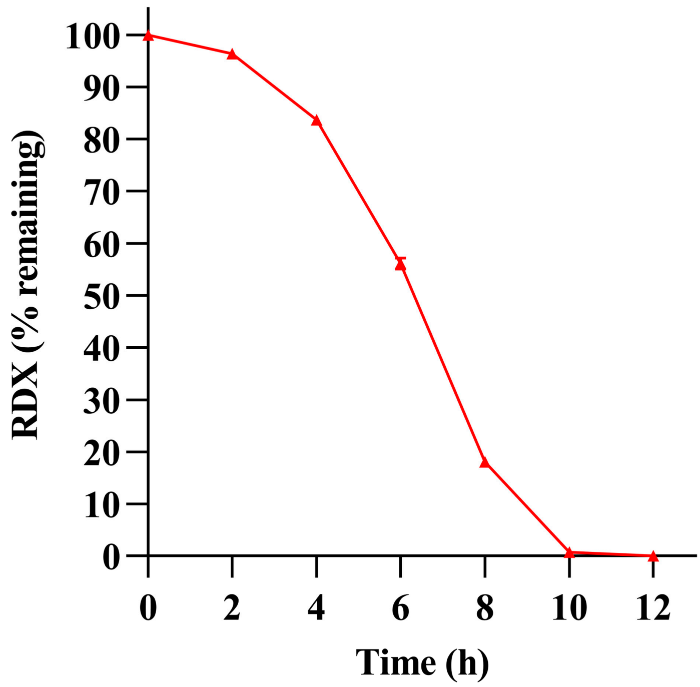

Figure S9. RDX degradation by 300 mg of *Rhodococcus* sp. strain DN22 cells in the medium utilizing RDX as an exclusive nitrogen source.

(A) RDX levels are shown in filled red triangles (n = 3).
