# Supplementary figures and images for: Multi-Omics Strategies to Investigate the Biodegradation of Hexahydro-1,3,5-trinitro-1,3,5-triazine in Rhodococcus sp. Strain DN22
Source: Microorganisms. 2023 Dec 30;12(1):76. doi: 10.3390/microorganisms12010076 (PMC10820124; doi:10.3390/microorganisms12010076)

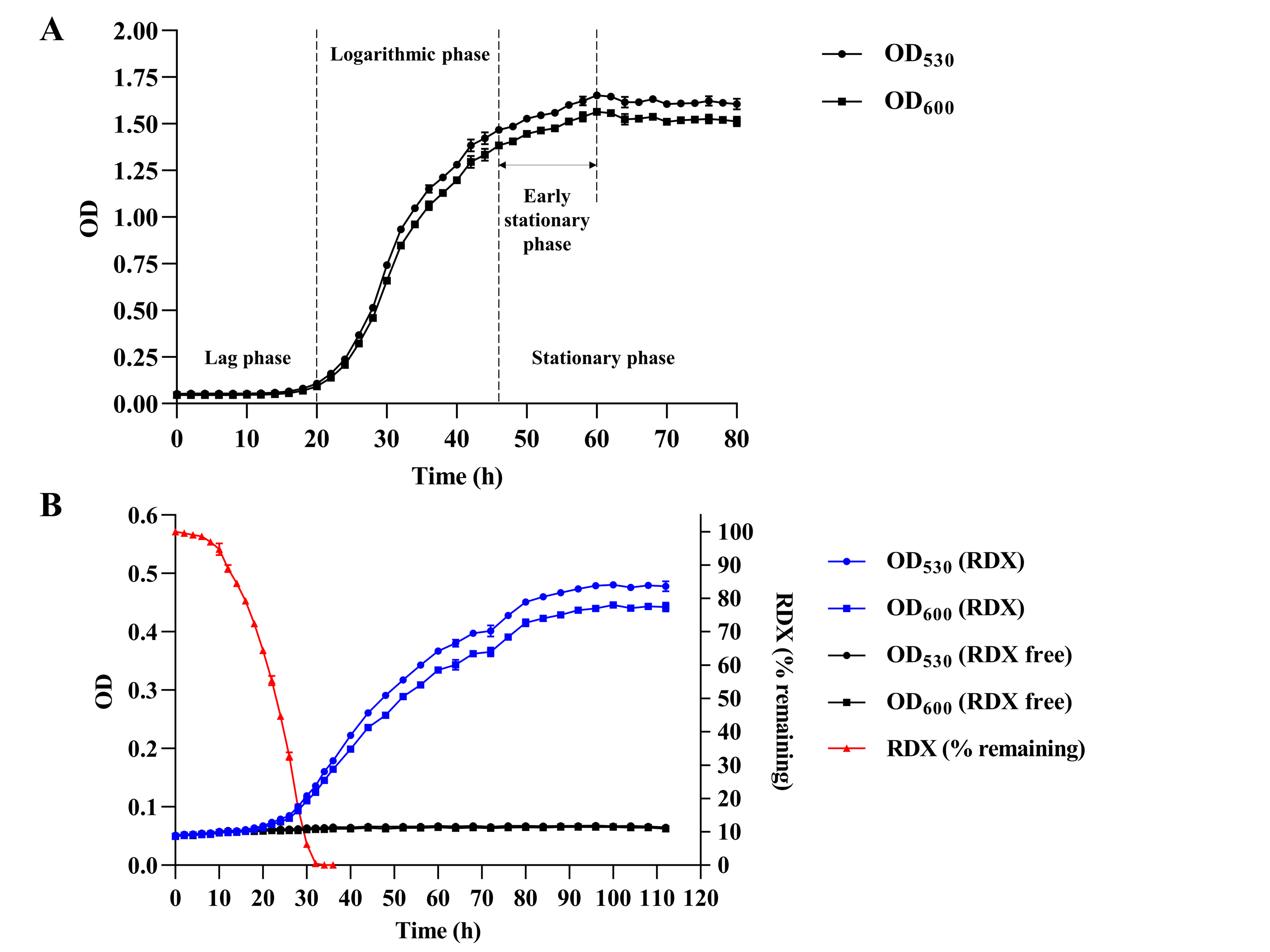

Supplement: Supplementary file 1 [file microorganisms-12-00076-s001.zip › Figure S1.tif]

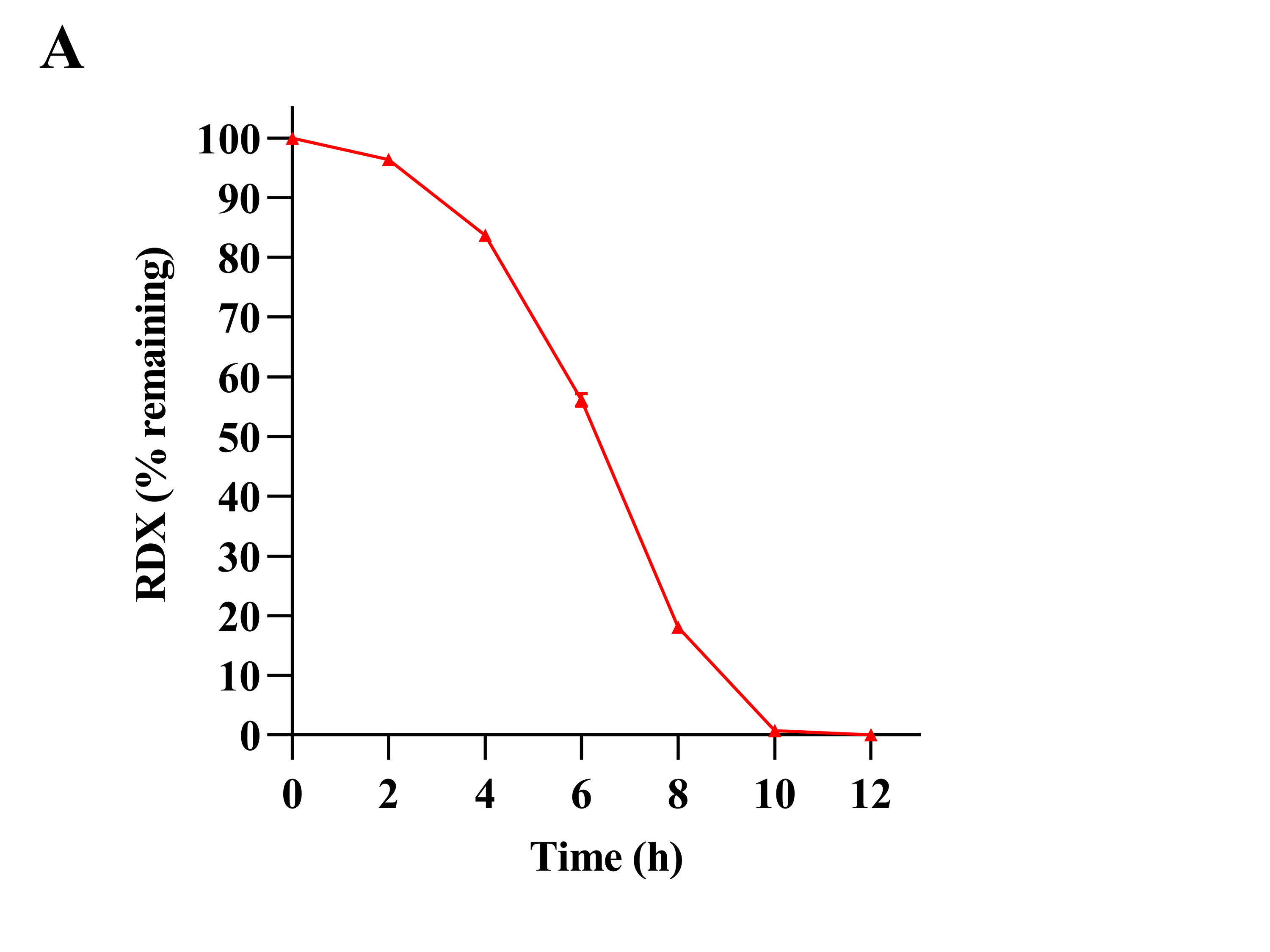

Supplement: Supplementary file 1 [file microorganisms-12-00076-s001.zip › Figure S9.tif]
